# Malate Transport and Metabolism in Nitrogen-Fixing Legume Nodules

**DOI:** 10.3390/molecules26226876

**Published:** 2021-11-15

**Authors:** Nicholas J. Booth, Penelope M. C. Smith, Sunita A. Ramesh, David A. Day

**Affiliations:** 1 College of Science & Engineering, Flinders University, GPO Box 5100, Adelaide, SA 5001, Australia; nick.booth@flinders.edu.au (N.J.B.); sunita.ramesh@flinders.edu.au (S.A.R.); 2 School of Life Sciences, La Trobe University, Bundoora, VIC 3083, Australia; p.smith3@latrobe.edu.au

**Keywords:** malate, metabolism, legume, nodules, nitrogen fixation

## Abstract

Legumes form a symbiosis with rhizobia, a soil bacterium that allows them to access atmospheric nitrogen and deliver it to the plant for growth. Biological nitrogen fixation occurs in specialized organs, termed nodules, that develop on the legume root system and house nitrogen-fixing rhizobial bacteroids in organelle-like structures termed symbiosomes. The process is highly energetic and there is a large demand for carbon by the bacteroids. This carbon is supplied to the nodule as sucrose, which is broken down in nodule cells to organic acids, principally malate, that can then be assimilated by bacteroids. Sucrose may move through apoplastic and/or symplastic routes to the uninfected cells of the nodule or be directly metabolised at the site of import within the vascular parenchyma cells. Malate must be transported to the infected cells and then across the symbiosome membrane, where it is taken up by bacteroids through a well-characterized *dct* system. The dicarboxylate transporters on the infected cell and symbiosome membranes have been functionally characterized but remain unidentified. Proteomic and transcriptomic studies have revealed numerous candidates, but more work is required to characterize their function and localise the proteins in planta. GABA, which is present at high concentrations in nodules, may play a regulatory role, but this remains to be explored.

## 1. Introduction

Legumes are able to form a symbiosis with soil bacteria, collectively termed rhizobia, that allows them to access N_2_ from the atmosphere. This process of symbiotic nitrogen fixation is an important contributor to the biological nitrogen cycle and an important component of sustainable agriculture, as it reduces the requirement for expensive nitrogen fertilizers and the pollution that can arise from their overuse. Housed within specialized organs, called nodules, on the roots of legume plants, rhizobia fix atmospheric N_2_ via the enzyme nitrogenase to produce ammonia and provide it to the plant in return for reduced carbon generated via photosynthesis. The nodules provide the microaerobic conditions that are required for synthesis and operation of nitrogenase, which is oxygen labile.

The establishment of the symbiosis involves signaling between the symbionts that results in the rhizobia moving through an infection thread derived from an invaginated plasma membrane and into the root cortex where the nodule has been initiated [1]. In response to unknown signals, the infection thread wall degrades and rhizobia enter cells in the immature nodule via invagination of the cell plasma membrane. The rhizobia, together with the enveloping host membrane, subsequently proliferate to fill the infected cell with symbiosomes, vacuole-like structures consisting of the plant-derived symbiosome membrane (SM) and the enclosed rhizobia [2,3,4,5]. Within the symbiosomes, the rhizobia differentiate into their symbiotic form, the bacteroid. The SM, while initially derived from the plasma membrane, becomes specialized as an interface between the bacteroid and its plant host, segregating the bacteroids from the plant cytoplasm and “protecting” them from any plant defense response. Movement of metabolites and signals across the SM is controlled via an array of transport proteins that are encoded by plant nuclear genes and inserted in the SM [3,6,7].

**Figure 1:**
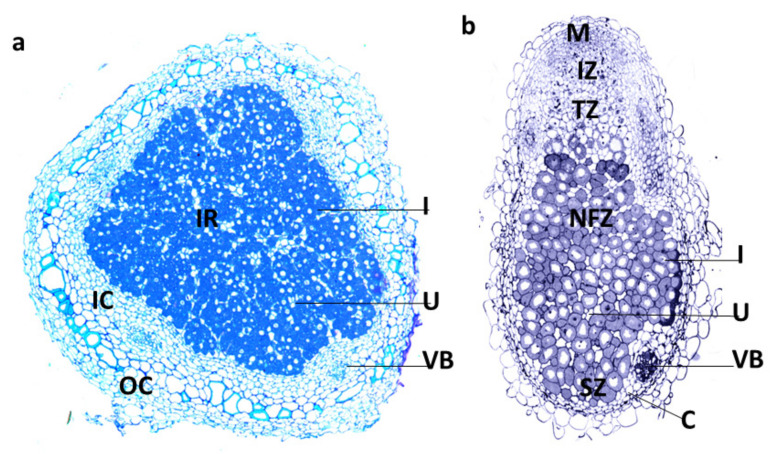
Structure of determinate (**a**) and indeterminate (**b**) nodules. a. Section of a determinate nodule from soybean. Determinate nodules do not have a persistent meristem. The central infected zone contains large infected cells (I) containing symbiosomes and smaller uninfected cells (U). This region is surrounded by the cortex including the inner cortex (IC) with vascular bundles (VB) and the outer cortex (OC). (**b**). Section of an indeterminate *M. truncatula* nodule. The nodule has a persistent meristem (M, often termed zone I) and continues to grow producing zones of different developmental stages including the infection zone (IZ, zone II), transition zone (TZ, interzone II-III), nitrogen fixation zone (FZ, zone IV) and senescence zone (SZ, zone V). The nitrogen fixation zone contains both infected cells (I) and uninfected cells. Vascular bundles (VB) are present in the nodule cortex (C). Images courtesy of Aleksandr Gavrin.

Nitrogen fixation has been studied extensively in a number of different legumes, including the model plants *Medicago truncatula* and *Lotus japonicus*, and important crops such as soybean (*Glycine max*) and pea (*Pisum sativum*). Nodules provide the microaerobic conditions required for the rhizobia’s nitrogenase enzyme to remain functional. Mature nodules are composed of an infection zone, which contains both infected and uninfected cells, surrounded by layers of cortical cells [1,8]. Nodules are generally considered as two distinct types, determinate and indeterminate (Figure 1). Determinate nodules, such as those on soybean and *Lotus japonicus*, are spherical and clearly divided into the central infected zone and the surrounding cortex. Within the infected zone, many of the cells contain symbiosomes with bacteroids that are mostly at approximately the same developmental stage [9], but this zone also has uninfected cells between the infected cells, which play important roles in N assimilation. Indeterminate nodules such as those on *Medicago truncatula* and *Pisum sativum*, on the other hand, have an elongated or branched shape and have a persistent meristem that remains throughout the life of the nodule. In branched nodules, each branch has a persistent meristem at its tip. Indeterminate nodules are segmented into distinct developmental zones: a meristematic zone at the tip, an invasion zone where the invading rhizobia are released, a transition zone where the rhizobia differentiate into bacteroids, a nitrogen fixation zone, and a zone of senescence closest to the root that develops as the nodule ages. In both types of nodule, nutrients are transported to the nodule via the vasculature, which terminates in the cortex. Symbiosomes in indeterminate nodules typically contain only a single bacteroid, while those in determinate nodules are larger and may contain many bacteroids. 

## 2. Nodule Metabolism and Provision of Carbon to the Bacteroids

The demand for fixed carbon in nodules is very large compared to the rest of the plant, mainly because of the need for C-skeletons during the assimilation of ammonium in the plant cells and the large energy costs of the nitrogenase reaction in the bacteroids:

N_2_ + 8H^+^ + 8e^−^ + 16ATP → 2NH_3_ + H_2_ + 16ADP + 16Pi.

Despite the fact that nodules constitute only a small fraction of the weight of a typical legume plant, they can consume more than 25% of the total photosynthate [10,11]. Studies with ^14^CO_2_ pulse-chase labelling of soybean have shown that recently fixed carbon in leaves is rapidly exported to the nodule as sucrose [12]. Similar experiments where nodules were incubated with ^14^CO_2_ demonstrate the activity of phosphoenolpyruvate carboxylase (PEPC) that uses metabolites derived from the breakdown of sucrose to produce organic acids in the plant cytosol that are subsequently assimilated by bacteroids [13]. Studies with isolated symbiosomes have shown that they are largely impermeable to sugars but take up organic acids like malate and succinate readily via a specific transporter [6]. Rhizobia mutants that are unable to metabolise organic acids produce Fix^-^ nodules, while those unable to metabolise sugars produce Fix^+^ nodules [14]. These organic acids also provide the carbon skeletons for the assimilation of ammonium, produced in the bacteroids, into amino acids or ureides to be exported out of the nodules to the rest of the plant [11,13,15].

Although the activity of many of the enzymes involved in conversion of sugars to organic acids is present in both infected and uninfected cells in determinate soybean nodules, the specific activity of the enzymes in the uninfected cells suggests that these cells make a greater contribution [11,16,17]. In the indeterminate nodules of pea, genes encoding nodule enhanced (ne)-sucrose synthase (SS), ne-malate dehydrogenase (MDH) and PEPC are expressed in both infected and uninfected cells [18] but the contribution of each cell type to organic acid production is not clear. The vascular parenchyma of the nodule also contributes to sucrose breakdown with ne-SS and PEPC transcripts localised in the vascular bundle of pea nodules [18] and MDH transcripts detected in cortex tissue containing vascular bundles in *L. japonicus* [19]. More work in this area is required to confirm the metabolic roles of the different cell types in the different legumes. 

**Figure 2:**
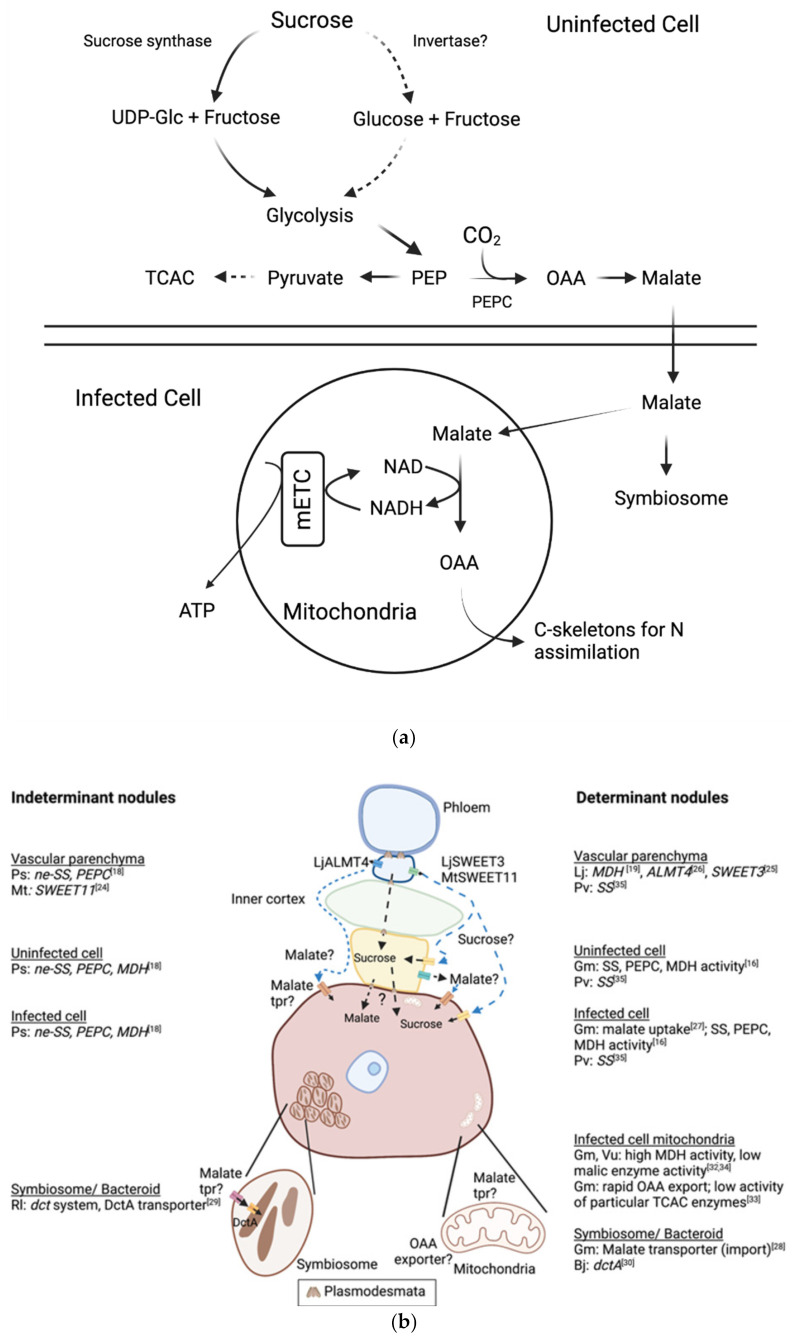
Nodule carbon metabolism. (**a**) The metabolism of sucrose to produce malate is likely to be in uninfected cells but may also occur in infected cells. In infected cells malate is transported to the symbiosome to support nitrogen fixation by bacteroids or to mitochondria, where it is used to generate ATP and the carbon skeletons required for nitrogen assimilation. Arrows with dashed lines indicate reactions that may occur but are unlikely to be significant in production of malate. PEP: phosphoenolpyruvate; PEPC: PEP carboxylase; OAA: oxaloacetate; TCAC: tricarboxylate cycle; mETC: mitochondrial electron transport chain. (**b**) Summary of gene expression, enzyme and transporter localisation in nodules and putative pathways for sucrose and malate movement within the nodule. Apoplastic routes are suggested by the presence of LjALMT4, LjSWEET3 and MtSWEET11 on the plasma membrane of nodule vascular parenchyma [24,25,26]. Malate transporters have been characterized on the infected cell plasma membrane [27] and symbiosome membrane [28] but the proteins encoding them have not been described. Malate is transported into bacteroids by a dicarboxylate transporter DctA, that is upregulated under symbiotic conditions [29,30]. Malate must be imported into the mitochondria but the transporter has not been described [31,32,33,34]. A sucrose importer may be present on infected and/or uninfected cell plasma membranes to support the activity of SS [35]. Symplastic routes are suggested by presence of plasmodesmata between cells in the nodule and studies with microinjection of Lucifer Yellow-CH and trafficking studies with GFP [20,21,22]. Ps: *Pisum sativum*, Mt: *M. truncatula*, Rl: *Rhizobium leguminosarum*, Lj: *L. japonicus*, Gm: *G. max*, Pv: *Phaseolus vulgaris*, Vu: *Vigna unguiculata*, tpr: transporter. Short dashed lines indicate possible pathways for malate movement. Long dashed lines indicate possible pathways for sucrose movement. Figure created with BioRender.com (accessed on 1 November 2021).

Sucrose imported to nodules and the dicarboxylates produced in the vascular bundles must reach the infected zone to be utilised in fuelling nitrogen fixation, but the pathway for this movement is not clear. Apoplastic and symplastic transport, or a combination of each, is possible. In determinate soybean nodules the presence of plasmodesmata means there are symplastic connections between all cell types and these connect phloem in the vascular bundle with both infected and uninfected cells within the infected region [20]. In indeterminate nodules, the presence of a symplastic pathway contributing to function of the mature nodule is not as obvious. In *Vicia faba*, plasmodesmata were identified between cells linking the vasculature with uninfected cells in the infected zone, but there were fewer connections to infected cells [21]. In *M. truncatula*, although symplastic connections are established and important during nodule development [22,23], there does not seem to be a symplastic connection between the interzone and the fixation zone in mature nodules [22]. What is clear is that sucrose, and possibly malate, could move symplastically from phloem at least part of the way to the infected cells, but based on activity of enzymes it is more likely that most sucrose is delivered symplastically to uninfected cells (Figure 2).

Transporters that potentially export dicarboxylate or sucrose from cells are expressed in the vascular parenchyma of nodules, suggesting that there is also an apoplastic route for carbon to reach the infected region of the nodule. Expression of SS in the infected region of *P. vulgaris* suggests there may be a sucrose importer on the infected and/or uninfected cell membranes [35]. Both *MtSWEET11* from *M. truncatula* [24] and *LjSWEET3* from *L. japonicus* [25] are expressed in the vasculature of nodules and the proteins they encode transport sucrose but not glucose (Figure 2). Since SWEET transporters allow movement of their substrate based on its concentration gradient, they have potential as exporters of sucrose that would move through the apoplast to infected or uninfected cells [24,25]. LjALMT4 is expressed in vascular parenchyma in *L. japonicus* nodules and heterologous expression in *Xenopus* oocytes indicated it could export malate, succinate and fumarate from these cells [26]. The fact that none of these transporters in themselves are essential for nitrogen fixation probably reflects redundancy in pathways for providing the bacteroids with an energy source through other transport proteins with similar functions or symplastic transport (Figure 2).

Isolated infected cells from soybean nodules actively take up malate, presumably via a specific transporter [27], and it is subsequently exported to the bacteroids via dicarboxylate transporters on the symbiosome membrane [28] and the bacteroid inner membrane [29,30,36,37], driven by the electrical and pH gradients across these membranes [6]. While the molecular identity of the bacteroid *dct* system is well established [38], neither the symbiosome nor the infected cell malate transporters have been identified. This is discussed below.

Experiments with isolated symbiosomes from different plants have shown that both malate and succinate are transported by the SM dicarboxylate transporter ([6] and references therein) and the transporter on infected cell membranes [27]. Nodules have high concentrations of both of these organic acids, most likely stored in the uninfected cells of the infected zone [11]. While it is clear that the malate is derived from sucrose via glycolysis, PEP-carboxylase and MDH [11,17], the source and fate of succinate is less clear. Succinate in most cells is produced in mitochondria, but its export from the mitochondria will disrupt the tricarboxylic acid cycle (TCAC) and it is generally not considered a major product of mitochondria. However, soybean and chickpea nodules, at least, also contain large quantities of malonate [39,40], which is a potent inhibitor of succinate dehydrogenase in the mitochondria. The role of malonate in nodules is not clear, although it may be a defense compound [40]. It is not a substrate for the symbiosome dicarboxylate transporter [28] and given that it is a respiratory poison, it is most likely contained in the vacuoles of uninfected cells. Its presence may explain the accumulation of succinate in nodules, but it appears that succinate is not required for nitrogen fixation in bacteroids: expression of a malate-specific transporter, MaeP, in *Sinorhizobium meliloti* Rm1021 *dct* mutants that are unable to transport C4-dicarboxylates, resulted in Fix^+^ nodules [41]. Taken together, all of the evidence to date indicates that malate is the predominate form of carbon that is supplied by the plant to support nitrogen fixation in the bacteroid. 

## 3. The Role of Mitochondria in Nitrogen-Fixing Nodules

Malate is a key substrate for mitochondrial respiration in many plant tissues and is also likely to be in nodule infected cells. Infected cells have a high energy demand to support transport processes and nitrogen assimilation, as well as leghemoglobin synthesis, and it is thought that mitochondrial oxidation of malate provides much of the required ATP (Figure 2). A number of studies with mitochondria isolated from nodules have shown that their metabolic capacity is substantially different from their counterparts in other tissues [31,32,33,34,42,43,44]. Nitrogen-fixing nodules contain large numbers of mitochondria, with about 12,000 per infected cell calculated from micrographs of soybean nodule cells [42]. These are found almost entirely around the periphery of the infected cells, next to airspaces [45], possibly tethered there by microtubules [46]. These studies have all been performed with determinate nodules, mainly from soybean or cowpea (*Vigna unguiculata*), and used entire nodules as the source of mitochondria. However, it is most likely that the mitochondria isolated were largely derived from infected cells [43]. These properties can be summarized as follows:

1. Oxygen levels in infected cells are maintained at very low levels (sub-micromolar) to allow operation of the nitrogenase enzyme [3,11]. Mitochondria from these cells have the capacity to respire under very low oxygen levels to supply the quantities of ATP estimated to be required to support symbiosome function and ammonia assimilation in infected cells [31,42,43]. The apparent oxygen affinity of cytochrome oxidase in nodule mitochondria is significantly greater than in those from roots [42].
2. They have very low alternative electron transport pathway activity, with virtually no alternative oxidase protein [47] and very low rates of cyanide-insensitive oxygen uptake. They also have very low rates of rotenone-insensitive malate uptake that is indicative of low internal alternative NADP(H) dehydrogenase (NDA) levels, although they oxidise external NADH rapidly [44]. This indicates that mitochondrial ATP production in infected cells is more efficient than in roots and other tissues.
3. They have very low malic enzyme activity, but very high malate dehydrogenase activity [32,34] and export oxaloacetate rapidly [33]. Together with low activity of other TCAC enzymes [33], this suggests that mitochondria of infected cells operate a truncated TCAC, oxidising principally malate to produce ATP and OAA for ammonia assimilation (Figure 2).

## 4. Malate Metabolism in Bacteroids

For malate (or succinate) to be sole carbon source for bacteroid respiration, pyruvate must be produced to drive the TCAC. Malic enzyme (ME) activity has been demonstrated in a number of different bacteroid species, from both determinate and indeterminate nodules [48,49,50,51]. ME oxidatively decarboxylates malate to pyruvate and, together with MDH, provides an effective means of generating both acetyl-CoA and OAA for bacteroid TCAC operation. In all species examined, bacteroids contain two ME forms, one of which is NAD^+^ specific and another which is NADP^+^ specific. Knockout mutants of the two isoforms in *S. meliloti* have shown that NAD-ME is essential for nitrogen fixation, but not NADP-ME [51,52]. These studies show that import of malate alone can provide the energy required for nitrogen fixation in bacteroids. 

## 5. Malate Transporters in Nodules

Given the key role of dicarboxylates, especially malate, in the nitrogen-fixing process, elucidating the mechanisms by which they are transported across cell and symbiosome membranes in nodules is crucial to our understanding of nodule function. 

The bacteroid C_4_-dicarboxylate system encodes three proteins, DctA, B and D. DctA is responsible for transport of dicarboxylates while the other proteins are involved in regulation of *dctA* expression. DctA is part of a wider transporter family that is present in both prokaryotes and eukaryotes [38]. It acts as a symporter with two protons transported for each malate, utilising the pH gradient across the bacteroid membrane [[6] see below]. 

While it is clear that dicarboxylate transporters exist on infected cell membranes and the SM, the molecular identity of these remains unknown. As mentioned above, it has been recently shown that succinate transport is not essential for nitrogen fixation [41], suggesting that malate is the most likely substrate for the SM dicarboxylate transporter in planta. By altering pH while maintaining total malate concentration, Udvardi and colleagues determined that the monovalent form of malate was the preferred substrate for the SM transporter [28]. Malate anion uptake into the symbiosome is affected by the rate of bacteroid respiration, energisation of the SM by a P-type ATPase, and phosphorylation of the transporter, probably via a calcium dependent protein kinase [53,54]. When comparing characteristics of the SM and infected cell dicarboxylate transport systems, many similarities arise. Both require energisation across their respective membranes to facilitate transport down a concentration gradient, and in both systems transport of malate and succinate competitively inhibit the transport of the other [27,28]. However, it is important to note that the transport of these two systems is distinct, as phthalonate potently inhibits transport by the SM dicarboxylate transporter but has little effect on dicarboxylate uptake by infected cells [27]. Both, phthalonate and cyanocinnamic acid are strong inhibitors of the SM transporter, distinguishing it from other plant and bacteroid dicarboxylate transporters and providing a pharmacological “signature” for it.

When considering possible candidates for the SM dicarboxylate transporter, it is important to understand the energetics of the symbiosome: proton pumping by the SM-ATPase into the symbiosome space provides a positive inside membrane potential and an acidic interior [18,55,56]. In this regard, the symbiosome resembles a vacuole and in fact symbiosomes largely replace the vacuoles in infected cells [4]. This makes tonoplast anion transporter families excellent candidates for the SM dicarboxylate transporter. Unfortunately, the nature of symbiosome uptake precludes using complementation of, for example, *mae1* deficient yeast or *E. coli* mutants to screen nodule cDNAs as candidates, since the transporter in question transports malate out of the plant cytosol (into the symbiosome) and therefore may not catalyse the uptake required for complementation. However, we can use available proteomic and transcriptomic data to identify potential candidates for further investigation [57,58,59].

Proteomic studies on isolated SMs have identified a number of putative transporters on the SM from pea, soybean, and *L. japonicus* [7,60,61], but few of these have been shown to act as anion transporters. Clarke and colleagues identified five members of the Nitrate/Peptide transporter Family (NPF) in soybean SM fractions [7]. Some NPF members can transport malate and other organic acids, but they are thought generally to operate as proton symporters [62] and, given the direction of the pH gradient across the SM, they are likely to transport compounds out of the symbiosome. However, there are some exceptions: in the non-legume *Alnus glutinosa* an NPF member, designated as DCAT1, localises to the symbiotic interface of infected nodule cells and was found to have dicarboxylate transport activity when expressed in *E. coli* and *Xenopus* oocytes [63]. More recently, some members of the NAXT subgroup of the NPF family have been shown to be anion excreters in roots, mainly of nitrate. For example, Arabidopsis AtNAXT1 apparently facilitates the uniport of one NO_3_^-^ per H^+^ pumped by the H^+^-ATPase [64], a mechanism reminiscent of the malate uniporter described in soybean symbiosomes (see above). A closer examination of the NPF proteins identified in the soybean SM proteome is clearly warranted.

Other prospective anion transporters have been identified in soybean SM fractions: these include five ABC transporters and four Voltage-Dependent Anion Channels (VDACs) [7]. ABC transporters are found in all known organisms and are capable of transporting a very wide range of substrates [65], including malate [66]. While ABC transporters share some characteristics with the SM dicarboxylate transporter, including phosphorylation-regulated transport [67], ABC transporters actively transport their substrates. In comparison, malate transport into isolated symbiosome appears to be passive, driven by the electrical potential across the SM [6,28]. The presence of VDAC proteins in SM fractions [60] is likely due to mitochondrial contamination of the SM fractions, since Wandrey and colleagues immunolocalised these VDACs in *L. japonicus* nodules and found they colocalised with mitochondria but not symbiosomes [68].

While no definitive candidates for the SM transporter have been identified in proteomic studies, transcriptomic data can be used to screen for more suitable candidates. Tissue–specific transcriptomic analyses of *M. truncatula* and *L. japonicus* [19,69,70] have been performed and allow identification of nodule enhanced transcripts. 

The tonoplast Dicarboxylate Transporter (tDT) family shares some similarities with the SM dicarboxylate transporter, transporting malate [71] and being inhibited by carbonyl cyanide m-chlorophenylhydrazone [72]. However, these proteins act as co-transporters, generally exchanging malate with citrate [72,73]. Additionally, no nodule-specific tDT isoforms are evident in published transcriptomic databases, so they are unlikely to be candidates for the SM dicarboxylate transporter.

SLow Anion Channel (SLAC) and the homologous (SLAH) proteins were originally annotated as dicarboxylate carriers due to sequence similarities with MAE1, a yeast dicarboxylate transporter, and may be involved in dicarboxylate transport in guard cells during stomatal opening [74]. Expression of Arabidopsis SLAC1, SLAH2, and SLAH3 in *Xenopus* oocytes generated anion currents when phosphorylated, with a preference for nitrate but some permeability to malate [75,76]. There is enhanced nodular expression of at least one member of this gene family in both *M. truncatula* [69] and *L. japonicus* [19,70], and they warrant further investigation for intracellular location and transport activity.

The published characteristics of the SM dicarboxylate transporter most closely represent those of the Aluminium-activated Malate Transporters (ALMT) family. This family was first identified in wheat where high aluminium concentrations caused an efflux of malate from the roots [77], but ALMTs have been found in a wide range of tissues from a number of different plant species, where they mediate organic acid transport across membranes. Some of these, such as AtALMT9, are located on the tonoplast where they catalyse malate uptake into the vacuole [78]. ALMTs transport a range of anions, display voltage-dependent gating and are regulated by phosphorylation [79], all characteristics shared with the SM transporter [28,54,80]. Takanashi and colleagues identified seven ALMT transcripts in *L. japonicus* and three were expressed in the nodule [26]. Of these, LjALMT4 shows nodule-specific expression, but it was expressed only in the vascular parenchyma cells. Other ALMT transcripts are also express in nodules of *L. japonicus* [81], soybean [82,83], and *M. truncatula* [69], but these remain uncharacterized and have yet to be localised.

## 6. The Role of GABA in Nodule Metabolism and Transport

γ-Aminobutyrate (GABA) is a non-protein amino acid that is ubiquitous across all kingdoms of life. This four carbon molecule regulates plant growth and development and its levels change in response to both abiotic and biotic stresses [84,85]. The many roles of GABA include maintaining cytosolic pH, acting as an osmolyte, participating in C/N metabolism, protecting against oxygen deficiency, scavenging reactive oxygen species (ROS), defense against pathogens and herbivory, and more recently acting as a signaling molecule [86,87]. In legumes, GABA is present at quite high concentrations in nodules compared to other amino acids but its function remains unclear. In isolated pea nodules, GABA was the second most abundant amino acid detected in bacteroids originating from different bacterial strains such as *Bradyrhizobium japonicum*, and *Rihizobium leguminosarum* [88,89,90]. Further, in pea plants, when nodulated roots were incubated with ^15^N_2_ for 30 min, a rapid labelling of GABA in the cytosol and bacteroid fractions of *R. leguminosarum* was detected [91].

GABA is synthesized in the cytosol by the decarboxylation of glutamate by glutamate decarboxylase (GAD) via the GABA shunt in normal cells. Under stress, the GABA shunt is upregulated and synthesized GABA is transported across the mitochondrial membrane by the GABA—permease [84,92,93]. In the mitochondria, GABA is converted to succinate that feeds into the TCA cycle maintaining energy production. Studies suggest that rhizobial bacteroids have a modified GABA shunt that lacks the GAD enzyme and in the nodules of snake bean and soybean, this finding has been supported by low activity and oxygen dependency [94,95]. In soybean, approximately 0.7 μmol g^−1^ of GABA was present in the nitrogen-fixing nodules while only 0.01 μmol g^−1^ GABA was detected in bacteroids from cowpea that showed very little GAD activity. The absence of GAD in the bacteroids suggests that the most likely source of GABA in the bacteroids is of plant origin and may be derived from branched chain amino acids supplied to the bacteroids [96]. Further, exogenous application of GABA (15 mM) to *M. truncatula* petioles doubled the concentration of GABA in the nodules, and increased nodule activity and N_2_ fixation [97]. Higher concentrations of GABA were detected in both phloem and nodules under normal conditions which increased when nodules were partially excised [97]. In several legumes such as alfalfa, lupin, cowpea and soybean, varying concentrations of GABA have been found in the phloem [98,99,100,101,102]. These observations suggest that GABA is phloem mobile, is translocated from shoot to root and nodules and may have a role in enhancing symbiotic N_2_ fixation. 

Similar effects of GABA on nitrate uptake have been observed in the non-legume *Brassica napus*, wherein GABA from the shoots was translocated to the roots and the uptake of nitrate (NO_3_^-^) during nitrogen deficiency was positively correlated with GABA concentrations in the phloem [103]. In Arabidopsis seedlings supplied with exogenous GABA (50 mM), similar effects on nitrogen metabolism have been observed under limited nitrogen conditions [104]. 

Of particular relevance to this review is the GABA regulation of ALMT proteins that are involved in transport of malate [85,105]. A putative GABA binding motif was identified on the ALMT family of proteins and GABA binding to aromatic amino acid residues in the motif was shown to negatively regulate malate efflux [105]. ALMTs have been characterized in the nodules of *L. japonicus*, and nodule enhanced transcripts have been detected in both *M. truncatula* and soybean but remain uncharacterized [26,106]. It is probable that GABA in the nodules exerts regulatory control of malate transport mediated by the ALMTs during N_2_ fixation. 

All of these observations suggest that GABA functions both as a metabolite and signaling molecule in legumes in response to stress and conditions that enhance nitrogenase activity. However, the physiological role of GABA in nodules remains unclear and future studies should explore the role and mechanism of GABA accumulation and regulation of ALMTs in the nodules. 

## 7. Conclusions

There is now a consensus that malate is the form of carbon that is supplied to rhizobia bacteroids in legume nodules to support nitrogen fixation. While we have a good general understanding of malate production and metabolism in nodules, there are several gaps. In particular, the spatial distribution of enzymes of carbohydrate and organic acid metabolism, and associated transport proteins, between the different compartments and cell types of nodules is uncertain, as is regulation of their expression. Single-cell transcriptomics and proteomics following laser capture of infected and uninfected cells, together with the new technique of spatial transcriptomics [107], will make this localisation and regulation clearer. The molecular identity of the malate transporters on infected cell and symbiosome membranes also remains to be elucidated, together with the inter-related role of GABA in nodules.

## Data Availability

Not applicable.
